# Moderating Effect of Chief Executive Officer Narcissism in the Relationship Between Corporate Social Responsibility and Green Technology Innovation

**DOI:** 10.3389/fpsyg.2021.717491

**Published:** 2021-10-20

**Authors:** Hailan Yang, Xiangjiao Shi, Shuo Wang

**Affiliations:** Business School, Shandong Jianzhu University, Jinan, China

**Keywords:** corporate social responsibility, CEO, narcissism, green technology innovation, heterogeneity

## Abstract

This study focuses on the impact of corporate social responsibility (CSR) on green technology innovation (GTI) of firms and the moderating influence of the chief executive officer (CEO) narcissism through the lens of stakeholder theory and upper echelons theory. This research deconstructs CSR into internal CSR and external CSR in order to reveal the effects of different types of CSR on GTI. Based on a sample of 1,745 firm-year observations from 349 Chinese-listed firms across sectors between 2014 and 2018, we find that the fulfillment of internal CSR has a significant positive impact on GTI. This relationship is strengthened when the CEOs are narcissistic. The external CSR has a significant negative impact on GTI and this relationship is strengthened by CEO narcissism. The major contribution of our study is that it provides a theoretical contribution to the existing literature by deconstructing CSR into internal and external CSRs and enriches the studies in the context of CSR from a point of view of the particular personality trait of a CEO.

## Introduction

It is widely observed that as the most fundamental and important organizational form in the contemporary economic system, firms greatly influence the economy, society, and environment. Empirical evidence largely supports the notion that on facing the external disturbance and unexpected events (Xiang et al., 2021), the positive engagement of firms in activities that enhance social value will suffer from a smaller magnitude of loss and would be more likely to maintain the stability following the shock (Huang et al., 2020). Firms that contribute both societal and environmental success and are financially viable are more likely to have a long-term survival and sustainable competitive advantage (Mellahi et al., 2016).

Corporate social responsibility (CSR) and green technology innovation (GTI) are two key driving forces for sustainable development and for the social value of firms (Lins et al., 2019; Satapathy and Paltasingh, 2019). The goal of CSR is to take into account the expectations of various stakeholders and encourage a positive impact on the economic, social, and environmental performance (Aguinis and Glavas, 2012). Existing research on CSR performance mainly focuses on its motivation and economic consequences (Guzzo et al., 2020). The theories used to explain the rationality of CSR mainly include agency theory, property right theory, resource-based theory, pyramid theory, stakeholder theory, corporate citizenship theory, corporate ethics development theory, and so on (Pan and Guan, 2021). More recently, researchers have focused more directly on two streams of CSR research. First, the focus shifts from the direct economic consequences of CSR to the study of the intermediate mechanisms (Hanh and Hien, 2020). The researchers have looked more at the interrelationship between CSR and other disciplines like, for example, the relationship between CSR and the R&D capability of the firm, investment efficiency, brand value, employee pride, and others (Clarkson et al., 2008). Among the research on the interrelationships, the vast majority of studies have explored how CSR promotes firm development through the support of stakeholders (Luo et al., 2018). Second, the recent research also shifts the attention more toward the further deconstruction of CSR. From the perspective of content and motivation of CSR, extant studies mainly divide CSR into internal vs. external CSR and mandatory vs. voluntary CSR, and their respective impact on firms and society (Luo et al., 2007). Specifically, we are concerned in this paper with internal vs. external CSR based on the stakeholder theory, which holds a unique perspective of the organizations and offers a diverse description of the everyday actions of a firm (Sulkowski et al., 2018).

Green technology innovation, as a combination of innovation-driven and green development concepts, yields environmental benefits. It is a significant strategic enabler to acquire justifiable and low-carbon economic development (Albort-Morant et al., 2018; Ilvitskaya and Prihodko, 2018). In the recent years, there has been a growing recognition for the need to emphasize GTI as a result of growing global problems, such as resource scarcity and environmental degradation. To date, scholars have begun to explore two new research areas of GTI. First, more management theories are applied to analyze the influencing factors on GTI, such as principal-agent theory, stakeholder theory, resource-based theory, and upper echelons theory. Second, scholars have gradually shifted focus from examining the direct impact of various factors on GTI to focusing on the moderating and mediating effects of multiple influencing factors on GTI and from the perspectives of external regulation and the organizations as a whole to the perspectives of internal initiative and individual executives. This paper deeply investigates the impact of the individual executive as a moderating variable on GTI and enriches the influencing factors of GTI at different levels.

The chief executive officer (CEO) is the highest-ranking corporate officer in charge of decision making in nearly every organization. Although the CEO is subjected to company and government, the role in making major decisions is self-evident (Shah et al., 2021). A CEO not only implements diverse managerial practices, but also plays an essential role in creating a vision for the entire organization, which shapes firm value, management direction, and the identity of the firm (Hart, 1992). Therefore, the CEO will influence the CSR activities of the firms and decide whether the firm will take an environmentally-friendly development path. According to the upper echelons theory, the psychological beliefs and personal traits of executives influence the strategic choice and the behaviors of the firm. CEOs with different psychological characteristics may make different choices on the choices of firms. The psychological factors of CEO affect the corporate social responsibility (Li et al., 2020). Among the psychological beliefs and personality traits of CEOs, narcissism occupies a prominent position (Olsen et al., 2014; Reina et al., 2014). Narcissistic CEOs have a stronger desire to have inflated self-views and have self-views reinforced than non-narcissistic counterparts (Chatterjee and Hambrick, 2007; Campbell et al., 2010). They tend to adopt audacious and extreme strategies to highlight their own images. From the psychological point of view, narcissism can make people obsessed with power and appreciation and may cause a CEO to have an eye-catching manner, which has a direct impact on the choices of the firms (Petrenko et al., 2016). So, will narcissistic CEOs take the initiative to fulfill CSR to satisfy their vanity? This question needs to be tested in this study.

A significant body of research has sought to find a link among CSR, CEO narcissism, and GTI. However, the findings are quite different. Some of the research on CSR–GTI relationship argued that firms actively engaging in CSR activities will stimulate the firms to take GTI measures to maximize the interests of the stakeholders (Cassiman and Veugelers, 2006; Bhattacharya et al., 2008; Dimitrova, 2020). However, some scholars have found that CSR activities do not improve GTI since considerable resources of the firms are devoted to maintaining various external relationships (Leuz and Oberholzer-Gee, 2006; Mehlum et al., 2006; Xie et al., 2015). In terms of the relationship between CSR and CEO narcissism, the positive view believes that the involvement in CSR initiatives is more affected by personal drivers and characteristics of the executive because such activities easily generate public attention and image reinforcement (Hambrick and Mason, 1984; Finkelstein and Hambrick, 1996; Sanders, 2001; Chatterjee and Hambrick, 2007; Hambrick, 2007; Weidenbaum and Jensen, 2009; Petrenko et al., 2016). Thus narcissistic CEOs tend to emphasize external CSR to enhance their public image and generate admiration (Al-Shammari et al., 2019). The negative view holds that narcissistic CEOs are blind and arrogant, indifferent to the views of the stakeholders, and ignore the maintenance of the relationship with the stakeholders. Therefore they will not actively fulfill CSR (Mccarthy et al., 2017; Sauerwald and Su, 2019). All these inconsistencies may be explained by the fact of the unclear measurement and subdivision of CSR.

According to the literature, two problems are to be further studied. First, CSRs with different motives have various influences on the choices of the firms. However, the existing literature mainly employs a rough measurement of CSR. Policies on the implementation of CSR by firms will not be well-targeted without clear deconstruction of CSR and comparative research. Second, although quite a few studies in the context of CSR are discussed from a psychological point of view, narcissism is less explored in the existing literature. Narcissism plays an important role during the process of the sustainable development of the firms, which easily generate public attention and image (Petrenko et al., 2016).

To solve these problems, this study examines the publicly traded Chinese A-share firms between 2014 and 2018. This study was conducted in the context of China since the exploration of the social value of the firms has attracted increasing interest from academics and the business community (Li and Zhang, 2010; Wang et al., 2018). The study makes several contributions. First of all, we provide a theoretical contribution to the existing literature by deconstructing CSR into internal CSR and external CSR based on the stakeholder theory. While there are a number of excellent studies based on this theory discussing the relationship between CSR and innovation, attempts at such deconstruction are not common since they largely interpreted the CSR as a whole. Deconstructing CSR into internal and external CSR can potentially explain the prior conflicting results in Xie et al. (2019) and Leuz and Oberholzer-Gee (2006) which concluded that CSR activities are negatively related to GTI. It is also helpful in clarifying the conflicting results regarding the relationship between CSR and CEO narcissism, such as the studies by Sauerwald and Su (2019) and Mccarthy et al. (2017) which found that narcissist CEOs will not actively fulfill CSR. In this study, we document the systematic differences between the internal CSR and external CSR when exploring the relationship among CSR, GTI, and CEO narcissism. Moreover, this deconstructing between the internal CSR and external CSR avoids the potential pitfall in the “one size fits all” recommendation that advocates engaging in all CSR activities will stimulate the firms to take GTI measures.

Second, we contribute to a deeper understanding of the managerial behavior in the Chinese firms by exploring how CEO narcissism impacts CSR-related decisions. CEOs play an essential role in decision-making and their personal traits affect the strategic choices. We relate their psychological belief and personality trait to the choices of the firm and highlight the role of CEO narcissism, which tends to be overlooked in the usual CSR research. It provides new insights into the research on the initiative of the CEOs to fulfill CSR from a psychological point of view. Narcissism as a psychological incentive may drive the CSR-related decisions of the CEO and thus reinforce or weaken the CSR–GTI relationship. This study introduces the CEO narcissism as a moderating variable and explores the role of this variable in the impact of CSR on GTI, which is an initiative in empirically testing the effect of CSR on GTI.

Third, this study explores the relationship among CSR, GTI, and CEO narcissism, which to the best of our knowledge has not been studied yet in the literature. It is conducted in the context of China, highlights the impact of CSR on GTI, and uses CEO narcissism as the moderating variable. Integrating the stakeholder theory and the upper echelons theory, we show that firm choices are not only influenced by the organizational-level factors but also by individual-level factors. In doing so, we enrich the literature on CSR and make further attempts to examine CEO narcissism as a significant factor impacting on the relationship between CSR and GTI.

## Theoretical Background and Study Hypotheses

### Theoretical Background

Based on the stakeholder theory, upper echelons theory, and researches on GTI, this section analyzes the influence of CSR on GTI and explores the moderating effect of CEO narcissism. Evidence shows that stakeholders prefer firms that are socially responsible because the firms that actively support CSR are supposed to be more reliable, and therefore their products are of higher quality (McWilliams and Siegel, 2001; Amos and Awuah, 2017). Luetkenhorst (2004) found firms could develop a number of benefits *via* adopting CSR strategies, such as cost savings, staff loyalty, healthy relationship with government, good reputation, positive consumer responses, and others. The involvement of diverse stakeholders in the management of the firms creates a friendly cooperation atmosphere and contributes to the success of the business. Thus stakeholder theory argues that satisfying the multiple needs and demands of the stakeholders is consistent with the interests of shareholders, which is the best way to maximize the value of the shareholders (Goethel et al., 2019; Mcdermott, 2020).

According to the upper echelons theory, the personal traits of the CEO affect the strategic choices. Different executives have different styles of management, which are closely related to firm performance. Narcissistic CEOs are bold, easily encouraged by social praise, and have strong political consciousness (Abhinav et al., 2018). As a result, they are eager to obtain more decision-making power to align the strategies of the firm with their preferences (Chatterjee and Hambrick, 2011).

Technology innovation plays a crucial role in achieving economic growth and environmental protection. GTI of firms can enhance the innovation ability of the area where they are located (D'Agostino and Moreno, 2019). Compared with the traditional technology innovation relying on capital and human resources and aiming for economic benefits, the GTI integrates economic performance with environmental protection (Thom and Sousa, 2016). Firms are facing the pressures of different stakeholders in terms of GTI. For example, the environmental regulations of the government and the pressures of public opinion impose restrictions on firms that pollute, and drive them to carry out GTI and reduce environmental pollution. The increasing environmental awareness and innovation ability of the employees promote the firms to enhance the awareness of green innovation, thereby improving the performance of GTI. The competition among the firms may also promote them to carry out GTI and gain the first mover advantage (Chang, 2016; Dangelico et al., 2017). Taking into account the attitudes of diverse stakeholders in the process of technological innovation, they can help the firm build a better social image and gain a higher market share (Talke and Hultink, 2010).

To sum up, this paper believes that fulfilling CSR to stakeholders is the key factor affecting the performance of GTI. CEO narcissism may affect CSR decisions, and then moderate the impact of CSR on GTI.

### Hypotheses Development

#### Corporate Social Responsibility and Green Technology Innovation

Distinct from other innovative maneuvers, GTI is a creative initiative that consists of unique processes and products (Xie et al., 2019). The high uncertainty and high cost involved often suppress the innovation of the firms. CSR is a self-regulating business model that helps a firm be socially responsible to itself, its managers, employees, customers, and the local population. CSR endeavors are concerned of giving back to society and satisfying the interests of the stakeholders. The better the social responsibility is fulfilled, the more demands of the stakeholders are taken into account; when the firm makes decisions, the more harmonious is the relationship between the firm and its stakeholders and the better are the image and the social status of the firm (Luetkenhorst, 2004; Bocken, 2015). Thus firms are more successful if they keep mutually beneficial relations based on trust with the stakeholders and thus have more resources for GTI (Clarkson et al., 2008; Cox and Wicks, 2011). Furthermore, CSR directly affects the very people who invest in the firm, therefore creating a cycle of benefit for both the firms and the community which requires investing firm resources for a payoff that is both distant and uncertain (Rangan et al., 2012). In this sense, firms with better CSR engagement attract higher quality investors and gain more financial support. To sum up, firms with better CSR plans obtain far better gains in business, have more faithful customers, and are generally much more profitable. As a result, firms are more likely to conduct innovation activities. We therefore pose the following hypothesis:

H1:The fulfillment of CSR has a positive impact on the performance of GTI.

#### Heterogeneous CSR and GTI

Based on the stakeholder theory, stakeholders are divided into market and nonmarket stakeholders (Driessen and Hillebrand, 2013). Market stakeholders are shareholders, customers, suppliers, and employees. This group of stakeholders has a vested financial interest in the successful implementation of business goals. The nonmarket stakeholders are outside of the organization, such as government and community and have no vested financial interest in the firm. Gallo et al. (2000) classified the CSR into two categories: internal social responsibility (the provision of satisfactory products or services to society, the creation of economic wealth, and the overall development of people within the business and ensuring the sustainability of the business) and external responsibility (an effort to correct damage to the good of society). In this sense, the internal CSR is accountable to market stakeholders, while external CSR is accountable to nonmarket stakeholders. In the process of GTI, the firms adopt internal and external CSR practices to satisfy the needs of the market and nonmarket. The literature provides the evidence that with the rise of education level and the impact of frequent health events, the increase in awareness and pressures from both the market and nonmarket stakeholders have necessitated firms to be more positive in facing and handling green environmental issues (Driessen and Hillebrand, 2013; Foo et al., 2019). For example, customers have greater requirements to ensure the products to conform to environmental protection and energy saving (Kiefer et al., 2017); Suppliers focus more on the environmental performance of their products to meet the assessment criteria of buyer firms. Thus supplier collaboration has a positive effect on the environmental performance and green product design (Mitra and Datta, 2014); the employees with higher education have a stronger awareness of sustainable development to ensure the firms to improve GTI (Zhang and Zhang, 2013). Firms that positively perform internal CSR will incorporate views of the market stakeholders into the firm strategy, which has a positive impact on GTI. As to the nonmarket stakeholders, the existing research shows government regulating authorities enforce the laws and regulations on the environmental strategies of the firms; the public media, an effective carrier of the information of the firms to outside, exposes behaviors which damage the environment to the public (Mao and Wang, 2019). To avoid the punishment of the government and pressures from the news media and to enhance the image of the firm in the eyes of the public, the organizations are reevaluating their manufacturing processes in response to pressures concerned with the eco-friendly well-being of the nonmarket stakeholders (Kiefer et al., 2017; Zimmerling et al., 2017). In sum, firms may improve their performance in GTI by fulfilling the internal CSR by paying attention to market stakeholders like customers, competitors, and employees. At the same time, firms will positively or are forced to carry out GTI by fulfilling their external CSR *via* focusing on nonmarket stakeholders like government, media, and the public. Therefore, we offer the following hypothesis:

H2a: The fulfillment of internal CSR has a positive impact on the performance of GTI.
H2b: The fulfillment of external CSR has a positive impact on the performance of GTI.

#### CEO Narcissism and GTI

According to the upper echelons theory, the personality traits of the CEO influence the strategic choices of the firms (Sarfraz et al., 2020a). Narcissistic CEOs have strong self-admiration and confidence in themselves. They prefer to engage in showboating events to pursue praise and media attention (Petrenko et al., 2016). When the firm faces strategic change, such as technological breakthrough, narcissistic CEOs tend to adopt bold and risky strategies to receive greater attention (Lindsay, 1977; Chatterjee and Hambrick, 2007, 2011). Entrepreneur risk behavior will increase corporate R&D investment and promote corporate sustainable development (Sarfraz et al., 2020b). Narcissistic CEOs have a sense of power and arbitrary, often underestimate the risk, overestimate the return, and prefer new products and technology innovation (Kashmiri et al., 2017). Thus, CEO narcissism will increase R&D investment of firms for the purpose of generating public attention (Campbell et al., 2010). In sum, the more a narcissistic CEO is, the more focus he will put on the external attention and the more likely he is to adopt radical and breakthrough innovation. This argument leads to the following hypothesis:

H3: CEO narcissism has a positive impact on the performance of GTI.

#### Moderating Effect of CEO Narcissism

As the key driver in engaging in CSR, the CEO has the highest decision-making power and the commitment to ensure the sustainable development of the firm. Narcissistic CEOs act decisively, pay attention to the maintenance of social relationships, and are more invested in socially responsible initiatives to draw more attention to themselves and achieve their need for reputation and fame (Mccarthy et al., 2017). Al-Shammari et al. (2019) argues that there is a positive correlation between CEO narcissism and CSR fulfillment since pursuing CSR activities is a means through which CEOs enhance their image and esteem. Narcissistic CEOs are more engaged in charity and donation as a way to enhance their moral feelings of superiority and to attract attention and praise (Petrenko et al., 2016). Narcissistic CEOs are eager to receive respect from market stakeholders, such as employees, suppliers, and customers, and honor and support from nonmarket stakeholders, such as government, society, and the public. Therefore, they will positively affect CSR fulfillment and place equal emphasis on both internal and external social responsibilities. In sum, the CEO narcissism may promote the fulfillment of internal and external social responsibilities, and positively moderate the impact of internal and external social responsibilities on GTI. Hence, we offer the following hypothesis:

H4: CEO narcissism positively moderates the impact of fulfillment of internal CSR on the performance of GTI.
H5: CEO narcissism positively moderates the impact of fulfillment of external CSR on the performance of GTI.

**Figure 1:**
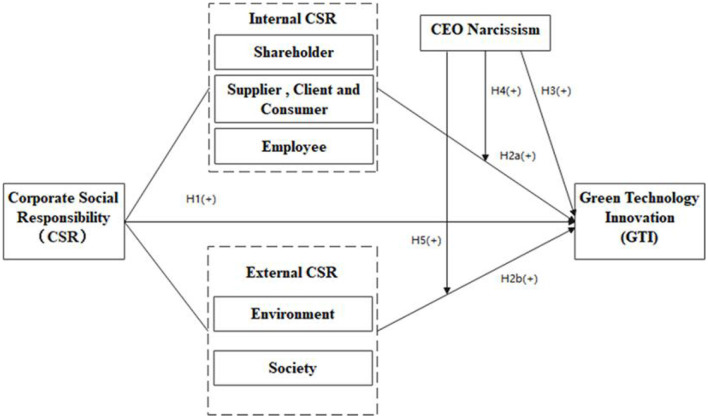
Conceptual model of the study.

Based on the above view, this paper puts forward a relevant study model. This model is shown in Figure 1, which integrates the relationships among CSR, GTI, and CEO narcissism (Figure 1).

## Sample and Variables

### Sample

Raw data were collected from archival and publicly available sources. The dataset of CSR rating scores of the listed firms of China was compiled from Hexun website (http://www.hexun.com), which provides financial information services as well as annual ratings for CSR strengths and concerns under several categories. Hexun rates more than 1,900 firms every year, which is sufficient to ensure the comprehensiveness of the data. The first-level indicators are clear and may distinguish the internal CSR and external CSR, consistent with the data collection requirements of this study. As standardized CSR data before 2013 are unavailable on Hexun, this study takes the firms listed on the main board of A-share of Shanghai and Shenzhen stock exchanges in China between 2014 and 2018. The preliminary samples are 9,625, excluding the following three types of firms: (1) Financial and insurance firms with different accounting standards and high-tech firms and other firms that cause mild pollution. Financial firms are excluded because one of the key objectives of this study is to investigate the influence of CSR on GTI. GTI is measured in the study by the green patent applications. The patent search results on Baiten.com show that the number of green patent applications by financial firms is basically 0, because the financial firms are rarely involved in GTI. Therefore financial firms are excluded from consideration in the study. (2) Firms with crucial missing data. This study uses CEO narcissism as a moderating variable, and uses five indicators to measure it. During the process of data collection, it is found that some firms do not have official websites, as a result of which their CEO narcissism data are unavailable. Thus these firms are excluded. (3) Firms with ST and ST^*^ (Chinese listed firms that are experiencing financial distress have a Special Treatment) between 2014 and 2018. In the data downloaded from Hexun website, there are firms subject to ST or ST^*^. ST is the abbreviation for Special Treatment in English, meaning “special treatment: with abnormal financial status or other conditions. Their abnormal financial or other conditions will affect the normal technological innovation and input in social responsibility. Thus they are excluded.

Regarding the sample period, this paper chooses 2014 as the starting year for the following reasons. First, CSR rating data of listed firms before 2014 are missing from the Hexun website. Second, in 2014, China completed the revision of the Environmental Protection Law of the People's Republic of China, which made the firms subject to stronger environmental constraints. This urged the firms to strengthen GTI to adapt to changes in the laws and environmental protection, so as to avoid strict legal sanctions. This paper chooses 2018 as the ending year of the sample period for the following reasons. First, during the data collection period, the patent information of some firms was unavailable on www.baiten.cn. In view of this situation, we searched patents in the CSMAR Patent Database and classified green patents according to the International Patent Classification (IPC) numbers. However, the patent data were only updated by 2018 in the CSMAR Patent Database. Second, the Report of the State Council of China on the Environment and the Achievement of Environmental Protection Targets in 2018 shows that the environmental protection of China made a breakthrough in 2018, which is a milestone year in the development of the ecological environmental protection of China. Therefore, from 2014 to 2018, the firms must have taken various energy-saving measures and actively carried out GTI to fulfill the tasks formulated for environmental protection in China. Third, the study conducted by Wang et al. (2018) looked into the change of the concerns of the Chinese government on environmental governance by analyzing the frequency of environment-related words, such as sustainability, climate, and pollution in the report on the work of the government. The study finds that the frequency of environment-related words in the report dropped sharply from 65 times in 2018 to 20 in 2019, which may be due to the impact of COVID-19. It is inferred that in 2019, the GTI of firms was also affected and hampered by COVID-19. Although the economy and the innovation of the firms revived gradually in the postepidemic era, this paper chooses 2014–2018 as the sample observation period to ensure the continuity of data and the representativeness of the study.

The final sample for the study was 1,745 firms and 13,960 sample observations were obtained. We collected other data from Baiteng (https://www.baiten.cn), Quanjing (http://www.p5w.net), Weibo of CEOs, corporate official websites, annual reports, and other social media sites. Stata 16.0 is used for statistical analysis.

### Variables and Measures

#### Dependent Variables

Green technology innovation is the dependent variable in our analysis. Nevertheless, there are many methods to measure GTI. These include green R&D expenditure, questionnaire method, energy consumption of new products, and economic model, each of which applies to specific situations. Based on the availability of data and the views put forward by Berrone et al. (2013), this paper uses the number of annual green patent applications as a measure of GTI. Technology patent is the most important output and index of innovations (Kemp, 2008); thus it is reasonable to use the number of green technology patents to measure the development of GTI. In order to search on green technology patents more efficiently, we searched the patent applications of listed companies on Baiteng, and used SOOPAT and Patent database as backup websites to screen the annual patent applications by using 13 keywords, such as “energy conservation,” “emission reduction,” “environmental protection,” “green,” “low carbon,” “emission,” “circulation,” “clean,” “pollution,” “environmental protection,” “energy consumption,” “noise,” and “sustainability. Considering the large difference in the number of green patent applications among samples, this study takes a natural logarithm for the number of green patent applications. Since there are samples with gti = 0, the result of taking logarithm for these samples is close to negative infinity; thus 1 is added to all gti before taking the logarithm.

#### Independent Variable

Corporate social responsibility is the independent variable. Following the studies of Wang et al. (2018), the authors downloaded the CSR ratings of the samples over the period from 2014 to 2018 from Hexun dataset. Hexun's social responsibility rating system consists of 56 indicators, including five dimensions of social responsibility, namely, shareholder responsibility, employee responsibility, suppliers, client, and consumer rights responsibility, environmental responsibility, and social responsibility. There are 13 level-2 sub-indicators and 38 level-3 sub-indicators under each of the five level-1 sub-indicators. Following the methods of prior studies, we divided the CSR into internal and external CSR from the perspective of heterogeneity. Shareholder responsibility, employee responsibility, and suppliers, client, and consumer rights responsibility fall under the internal CSR, while environmental responsibility and social responsibility fall under the external CSR. In this study, the two categories of CSR are separately totaled up, obtaining the total scores for internal and external CSR. Given the different scores of CSR, the natural logarithms are taken for the scores of the overall CSR, and the internal and external CSR. Details of Hexun CSR index construction are illustrated in Table 1.

**Table 1:** Hexun (HX) index system (http://www.hexun.com).

	**HX level-1 sub-indicators**	**HX level-2 sub-indicators**	**HX level-3 sub-indicators**
Internal CSR	HXSH (30%)	Earnings (10%)	Return on equity (2%) Return on total assets (2%) Profit margin of main business (2%) Rate of return on cost (1%) Earnings per share (2%) Undistributed profit per share (1%)
		Solvency (3%)	Quick ratio (0.5%) Current ratio (0.5%) Cash ratio (0.5%) Equity ratio (0.5%) Asset-liability ratio (1%)
		Returns to shareholder (8%)	Ratio of dividends to equity (2%) Dividend pay-out ratio (3%) Ratio of dividends to distributable profits (3%)
		Credit approval (5%)	Number of penalties imposed by the exchange on the company and relevant responsible persons (5%)
		Innovation (4%)	Product development expenditure (1%) Technological innovation concept (1%) Number of technological innovation projects (2%)
	HXST (15% in common industries, 10% in consumption industries)	Performance (5%)/(4%)	Per capita income of employees (4%)/(3%) Employee training (1%)/(1%)
		Safety (5%)/(3%)	Safety inspection (2%)/(1%) Safety training (3%)/(2%)
		Caring for employees (5%)/(3%)	Employee caring consciousness (1%)/(1%) List of members of caring for employees (2%)/(1%) Consolation money for employees (2%)/(1%)
	HXC (15% in common industries, 20% in consumption industries)	Product quality (7%)/(9%)	Quality management awareness (3%)/(5%) Certificate of quality management system (4%)/(4%)
		After-sales service (3%)/(9%)	Customer satisfaction survey (3%)/(4%)
		Integrity (5%)/(7%)	Fair competition among suppliers (3%)/(4%) Anti-bribery training (2%)/(3%)
External CSR	HXE (20% in common industries, 30% in manufacturing industries, 10% in service industries)	Environmental management (20%)/(30%)/(10%)	Environmental protection consciousness (2%)/(4%)/(2%) Environmental management system certification (3%)/(5%)/(2%) Investment in environmental protection (5%) /(7%)/(2%) Number of pollutant discharge types (5%)/(7%)/(2%) Number of energy-saving measure types (5%)/(7%)/(2%)
	HXS (20% in common industries, 10% in the manufacturing industries, 30% in service industries,)	Contribution value (20%)/(10%)/(30%)	Ratio of income tax to total profits (10%)/(5%)/(15%) Public donation amount (10%)/(5%)/(15%)

#### Moderating Variable

The chief executive officer narcissism is the moderating variable. Based on the study of Han and Li (2009), this study uses the president or the general manager to replace the CEO for firms without CEOs. Given the situation of this study and the four dimensional self-narcissism of Emmons, five indicators are selected to measure CEO narcissism (Chatterjee and Hambrick, 2007). The four-dimensional relationship between the five measurement indicators and the narcissistic personality inventory (NPI) is shown in Table 2.

**Table 2:** Relationship between CEO narcissism indicators and four dimensions of NPI.

**Narcissistic Personality Inventory (NPI)**		**The indicators and characteristics of CEO narcissism for the study**				
Four dimensions	Characteristics	Indicator 1: Prominence of the CEO in press releases	Indicator 2: Number of articles of CEO on social platforms	Indicator 3: CEO's use of first-person singular pronouns in interviews	Indicator 4: Prominence of the CEO's photograph in the annual report	Indicator 5: Number of CEO photos in the official Weibo account
Authority	I have the final say in everything and I lead everything	I am the core person of the firm and I should be the center		All achievements are under my leadership	I'm at the heart of the company	I am the top leader and the firm should center around me
Exhibitionism/ Self-sufficiency	I admire myself in the mirror and feel I am excellent	I enjoy the internal and external praise	My views are correct and others should listen to me	I am the core leadership and lead to the success	I am attractive	I am the leading and the most important person in the firm
Superiority/ Vanity	I hope to be superior to others	I am special and should deserve attention	Others should study hard and follow my opinions	I represent the whole firm	I'm the head of the firm and the publicity should focus only on me	I'm very important and should be vigorously publicized
Power desire	I need to be awed and am jealous of others' achievements	I should deserve a lot of coverage		I am in charge of the firm and any achievement is due to my involvement	My importance should be highlighted	The firm should highlight my importance and let more people know me

Indicators 1, 2, 3, and 5 are based on the studies of Wu and Gong (2018), while Indicator 4 draws on the studies of Sauerwald and Su (2019) and Al-Shammari et al. (2019). The detailed explanations of the five indicators are as follows. Indicator 1: Prominence of the CEO in press releases means the proportion of reports about CEO in the total number of reports on the official website of the company; Indicator 2: Number of articles of CEO on social platforms refers to the number of the original articles of CEO's on social platforms, such as Weibo; Indicator 3: The use of first-person singular pronouns by the CEO in interviews means the proportion of CEOs using the first person singular (I) in the use of first person (I/we) in Quanjing's interview with CEOs; Indicator 4: Prominence of the photograph of the CEO in the annual report is the proportion of the photo of the CEO in the photo page of the annual report. The proportion is scored based on the four-point scale (4 for a photo of the CEO alone in which the CEO occupies more than half of the page, 3 for a photo of the CEO alone in which the CEO occupies less than half of the page, 2 for a group photo, and 1 for no photos); Indicator 5: Number of CEO photos in the official Weibo account is the number of photos of the CEO appearing in the official account of the Weibo of the firm in that year. In order to measure the degree of CEO narcissism, the five indicators are standardized and the arithmetic mean is taken based on the previous literature.

#### Control Variables

We control for several factors known to affect GTI. The following variables are controlled based on the studies of Yu et al. (2019). (1) The area where the firm is located. The development level of the area, where the firm is located, directly affects the level of scientific and technological innovation. Economically advanced areas with preferential policies pay more attention to green environmental protection and scientific and technological innovation, and the firms will have strong GTI ability. (2) Firm nature. Compared with the most privately-owned firms, state-owned firms have stronger economic strength and sense of response to policies and stronger ability of GTI. (3) Industry. Compared with the light-polluting industries, the heavy-polluting industries cause more serious damage to the environment and shoulder greater responsibility to protect the environment, and have a strong sense of GTI. (4) Firm size. The size of firms directly affects the R&D investment. The large firms with abundant assets have stronger GTI ability and invest more in R&D. (5) Firm performance. The financial performance of a firm directly affects its R & D investment. Profitable firms have the economic basis for R&D and have stronger GTI ability. (6) The proportion of the technical staff in the firm. The number of technical staff in a firm affects the output of technological achievements. Firms with a high proportion of technical staff pay more attention to R&D and have stronger GTI ability. The variables are defined as shown in Table 3.

**Table 3:** Variable definitions and measurement.

**Variable type**	**Variable name**	**Variable symbol**	**Variable definition**
Explained variable	Green technology innovation	gti	ln (Number of green patent applications+1)
Core independent variable	Corporate social responsibility	csr	ln (Social responsibility rating scores of listed companies on Hexun)
	Internal corporate social responsibility	incsr	ln (Internal social responsibility rating scores of listed companies on Hexun)
	External corporate social responsibility	excsr	ln (External social responsibility rating scores of listed companies on Hexun)
Moderating variable	CEO narcissism	ceonar	The arithmetic mean of five measurement indicators after standardization
Control variable	Area of the enterprise	area	0-1 variable, 1 for East China, otherwise 0
	Enterprise nature	nature	0-1 variable, take 1 if the firm is state-owned
	Enterprise industry	industry	0-1 variable, take 1 if the firm is a heavy-polluting firm
	Enterprise size	size	ln (Total assets)
	Enterprise profitability	pro	Total profit/operating income
	Proportion of technical staff	tsrat	Number of technical staff/total employees

#### Research Model

In order to test H1, that is, the impact of CSR on GTI, we set up the regression model (1).


(1) $$\begin{array}{l} gti_{it}=\alpha +\beta_{1}csr_{it}+\beta_{2}area_{i}+\beta_{3}nature_{i}+\beta_{4}industry_{i}\\ \;+\beta_{5}size_{it}+\beta_{6}pro_{it}+\beta_{7}tsrat_{it}+\varepsilon_{it} \end{array}$$

Where, the dependent variable gti is the green technology innovation index of the firm, the independent variable is csr that represents the corporate social responsibility index, and ε represents the stochastic error term of the model, and the rest are control variables.

Based on the heterogeneity of CSR, CSR is divided into internal and external CSR. In order to test H2a and H2b, that is, the impact of internal CSR and external CSR on GTI, we construct the following regression model (2).


(2) $$\begin{array}{l} gti_{it}=\alpha +\beta_{8}incsr_{it}+\beta_{9}excsr_{it}+\beta_{10}area_{i}+\beta_{11}nature_{it}\\ \;+\beta_{12}industry_{i}+\beta_{13}size_{it}+\beta_{14}pro_{it}+\beta_{15}tsrat_{it}+\varepsilon_{it} \end{array}$$

Where, the dependent variable gti is the green technology innovation index of the firm, the independent variables are incsr (internal CSR index) and excsr (external CSR index), and ε represents the stochastic error term of the model, and the rest are control variables.

In order to test H3, H4, and H5, that is, the moderating effect of CEO narcissism on the main effect and the impact of CEO narcissism on GTI, a regression model (3) is set up.

As tested by the variance inflation factor (VIF), the interaction terms of incsr and ceonar, excsr and ceonar show serious multicollinearity with their low-order terms. To solve the serious multicollinearity, we referred to the studies of Xie (2010) and centered the low-order terms: The incsr, excsr, and ceonar are subtracted, respectively by their sample means and then the interaction terms are constructed, and the lower-order terms after subtracting the sample means are substituted into the regression equation, namely:


(3) $$\begin{array}{r} c_{-}incsr\;=\;incsr-\bar{incsr}\\ c_{-}excsr\;=\;excsr-\bar{excsr}\\ c_{-}ceonar\;=\;ceonar-\bar{ceonar}\\ gti_{it}=\alpha +\beta_{16}c_{-}incsr_{it}|+\beta_{17}c_{-}excsr_{it}+\beta_{18}c_{-}ceonar_{it}\\ +\beta_{19}interact_{it}+\beta_{20}interact1_{it}+\beta_{21}area_{i}+\beta_{22}nature_{i}\\ +\beta_{23}industry_{i}+\beta_{24}size_{it}+\beta_{25}pro_{it}+\beta_{26}tsrat_{it}+\varepsilon_{it} \end{array}$$

where gti is the dependent variable, interact and interact1, respectively represent the interaction terms of CEO narcissism with internal CSR and external CSR after subtracting the sample means, c_incsr, c_excsr, c_ceonar, interact, and interact1 are independent variables, and ε represents the stochastic error term of the model, and the rest are control variables.

## Empirical Analysis and Results

### Descriptive Statistics

Descriptive statistics are provided in Table 4. The mean GTI is 1, the standard deviation of GTI is 1.03, the minimum GTI is 0, and the maximum GTI is 7.15. This shows that there is a huge difference in GTI capability, and such capability needs improvement. The mean CSR is 3.61, the standard deviation CSR is 0.39, the minimum CSR is −1.11, and the maximum CSR is 4.61. This shows that there is a huge difference in the fulfillment of corporate social responsibility and improvement is needed. The mean internal CSR is 2.95, the standard deviation of internal CSR is 0.56, the minimum internal CSR is −0.34, and the maximum internal CSR is 4.09. This shows that there is a difference in corporate social responsibility performance and improvement is needed. The mean external CSR is 3.04, which is higher than the internal CSR; the standard deviation of the external CSR is 0.30, the minimum external CSR is 0, and the maximum external CSR is 3.97. This shows that the difference of external CSR is small, and the dispersion degree is lower than the internal CSR. Compared with the fulfillment of internal CSR, enterprises prefer to fulfill external CSR, as the latter can expand the market and build a good corporate image. The mean CEO narcissism is 0.059, the standard deviation of CEO narcissism is 0.28, the minimum CEO narcissism is 0, and the maximum CEO narcissism is 4.89. This shows that there is a huge difference in CEO narcissism among enterprises.

**Table 4:** Descriptive statistics.

**Variable**	**Obs**	**Mean**	**Std. Dev**.	**Min**	**Max**
Gti	1,745	1	1.03	0	7.15
Csr	1,745	3.61	0.39	−1.11	4.61
Incsr	1,745	2.95	0.56	−0.34	4.09
Excsr	1,745	3.04	0.30	0	3.97
Ceonar	1,745	0.059	0.28	0	4.89
Area	1,745	0.55	0.50	0	1
Nature	1,745	0.66	0.47	0	1
Industry	1,745	0.67	0.47	0	1
Size	1,745	24.17	1.60	17.03	27.67
Pro	1,745	0.11	0.30	−2.21	4.21
Tsrat	1,745	0.16	0.12	0	0.74

From the perspective of the main control variables, there are great differences in enterprise size and profitability, while the difference in the proportion of technical staff is relatively small. Nature is 0–1 variable, and the mean nature is 0.66. This shows that more than half of the enterprises are state-owned. Industry is 0–1 variable, and the mean industry is 0.67. This shows that more than half of the enterprises are high-polluting. The standard deviation of the area is 0.50, and the mean area is 0.55. This shows that most enterprises are located in East China.

### Correlation Analysis and VIF Test

Correlations and VIF tests of the main variables are provided in Table 5, intending to preliminarily test the correlation and check for the presence of multicollinearity. The results show that the absolute value of the correlation coefficient is generally lower than 0.4 (except the coefficient of external CSR and internal CSR, which is 0.527). To further verify there is no multicollinearity, the VIF test was conducted in this study. All variables have VIF <2, and the mean VIF is 1.25, indicating that multicollinearity is not a problem.

**Table 5:** Correlations and VIF test.

**Variables**	**Gti**	**Incsr**	**Excsr**	**Area**	**Nature**	**Industry**	**Size**	**Pro**	**Tsrat**	**VIF**
gti	1									
incsr	0.110***	1								1.75
excsr	0.011	0.527***	1							1.45
area	−0.112***	−0.145***	−0.004	1						1.09
nature	0.03	−0.023	0.076***	0.149***	1					1.15
industry	0.038	−0.179***	−0.079***	0.161***	0.053**	1				1.09
size	0.204***	0.195***	0.189***	−0.022	0.314***	0.055**	1			1.19
pro	−0.062***	0.385***	0.089***	−0.041*	−0.002	−0.145***	0.060**	1		1.21
tsrat	0.069***	0.108***	0.093***	−0.148***	0.062***	−0.150***	0.135***	0.045*	1	1.07

***p < 0.01, **p < 0.05, *p < 0.1.

The correlations and VIF test between the dependent variable and the core independent variables in the moderating effect are provided in Table 6. The absolute value of the correlation coefficients is generally lower than 0.5 (except the coefficient of the internal and external CSR, which is 0.527). All variables have VIF <2, and the mean VIF is 1.33, indicating that multicollinearity is not a problem in the moderating effect model.

**Table 6:** Correlations and VIF test.

**Variables**	**Gti**	**C_incsr**	**C_excsr**	**C_ceonar**	**Interact**	**Interact1**	**VIF**
gti	1						
c_incsr	0.110***	1					1.40
c_excsr	0.0110	0.527***	1				1.39
c_ceonar	0.148***	0.042*	0.0240	1			1.22
Interact	0.108***	−0.081***	−0.0270	0.260***	1		1.25
Interact1	0.0140	−0.0250	−0.051**	0.411***	0.431***	1	1.40

***p < 0.01, **p < 0.05, *p < 0.1.

### Empirical Tests

#### Model

In this study, mixed effect, fixed effect, and random effect were tested separately before conducting panel data regression to select the benchmark model. In the comparison between the mixed regression and fixed effect regression, the F statistic = 9.960, *p* = 0.000, which is considered that the fixed effect regression is significantly better than the mixed regression. In the comparison between mixed regression and random effect regression, the Lagrange multiplier (LM) statistic (chibar2) = 1400.100, *p* = 0.000, which is considered that the random effect regression is better than the mixed regression. In the comparison between fixed effect regression and random effect regression, the chi2 statistic of Hausman test = 9.280, *p* = 0.055 < 0.1, so the fixed effect model was used. Since the variables, such as nature, area, and industry remain unchanged over time, the REGHDFE regression was used. The Hausman test for the REGHDFE regression and the random effect regression was also used in this paper, with *p* = 0.000. Since the REGHDFE regression does not report the regression results of intercept terms and categorical variables, in order to observe their regression results, this paper also carried out mixed effect regression as a reference, and the regression coefficient and significance level of the two effects are basically consistent.

#### Main Effect Test

The regression results of the main effect are provided in Table 7, including Panel data mixed effect regression and REGHDFE regression.

**Table 7:** Results of mixed effect regression and REGHDFE regression.

	**(1)**	**(2)**
**Variables**	**gti**	**gti**
Csr	0.163***	0.163**
	(2.61)	(2.47)
Area	0.156***	Control
	(3.24)	
Nature	−0.055	Control
	(−1.03)	
Industry	0.104**	Control
	(1.97)	
Size	0.127***	0.127***
	(6.95)	(7.76)
Profitability	−0.314***	−0.314***
	(−5.87)	(−3.73)
Tsrat	0.310	0.310
	(1.40)	(1.47)
Constant	−2.658***	−2.539***
	(−6.18)	(−6.39)
Observations	1,745	1,745
R–squared	0.061	0.061
F test	0	0
r2_a	0.0568	0.0568
F	16.11	22.92

***p < 0.01, **p < 0.05.

In the REGHDFE regression of the main effect, the effect of CSR on GTI is positive and significant (β = 0.163, *p* < 0.05). The results are consistent with the mixed effect regression, which provide strong support for Hypothesis 1.

#### Further Analysis Based on Heterogeneous CSR

The regression results of heterogeneous CSR and moderating effect are provided in Table 8, including mixed effect regression and REGHDFE regression of heterogeneous CSR, mixed effect regression, and REGHDFE regression of the moderating effect.

**Table 8:** Mixed effect regression and REGHDFE regression of heterogeneous CSR, mixed effect regression, and REGHDFE regression of moderating effect.

	**(3)**	**(4)**	**(5)**	**(6)**
**variables**	**gti**	**gti**	**gti**	**gti**
Incsr	0.319***	0.319***		
	(6.03)	(5.67)		
Excsr	−0.359***	−0.359***		
	(−3.22)	(−3.81)		
C_ceonar			0.524***	0.524***
			(5.70)	(5.70)
Interact			1.045***	1.045***
			(4.24)	(4.24)
Interact1			−1.515***	−1.515***
			(−3.33)	(−3.33)
Area	0.130***	Control	0.131***	Control
	(2.71)		(2.65)	
Nature	−0.030	Control	−0.027	Control
	(−0.57)		(−0.51)	
Industry	0.112**	Control	0.136***	Control
	(2.14)		(2.61)	
Size	0.125***	0.125***	0.115***	0.115***
	(6.93)	(7.74)	(7.15)	(7.15)
Profitability	−0.442***	−0.442***	−0.419***	−0.419***
	(−7.23)	(−5.07)	(−4.87)	(−4.87)
Tsrat	0.330	0.330	0.365*	0.365*
	(1.53)	(1.58)	(1.77)	(1.77)
c_incsr			0.329***	0.329***
			(5.91)	(5.91)
c_excsr			−0.375***	−0.375***
			(−4.05)	(−4.05)
Constant	−1.888***	−1.761***	−1.914***	−1.779***
	(−4.21)	(−4.31)	(−4.97)	(−4.80)
Observations	1,745	1,745	1,745	1,745
R-squared	0.075	0.075	0.096	0.096
F test	0	0	0	0
r2_a	0.0707	0.0707	0.0906	0.0906
F	17.41	24.01	16.56	20.46

***p < 0.01, **p < 0.05, *p < 0.1.

Most of the existing studies only explored the integrated indicator of CSR. On this basis, this study further explored the effect of internal and external CSR on green technology innovation (GTI). In column (4) of Table 8, the effect of internal CSR on GTI is positive and significant (β = 0.319, *p* < 0.001). The results are consistent with the mixed effect regression, which provide strong support for Hypothesis 2a.

The effect of external CSR on GTI is negative and significant (β = −0.359, *p* < 0.001) and the results are consistent with the mixed regression results. The negative sign indicates that the higher the degree of fulfillment of external CSR, the higher is the degree of constraint on GTI. The results do not support Hypothesis 2b. The reason may be that enterprises pay too much attention to external CSR, which leads to the transfer of resources to the maintenance of external social relations and market expansion. Having a good social image and being protected by the government and the public, these enterprises may still survive with no or less technology innovation.

#### Moderating Effect Tests

In column (6) of Table 8, the effect of ceonar on GT is positive and significant (β = 0.524, *p* < 0.001). The results are consistent with the mixed effect regression, which provide strong support for Hypothesis 3.

**Figure 2:**
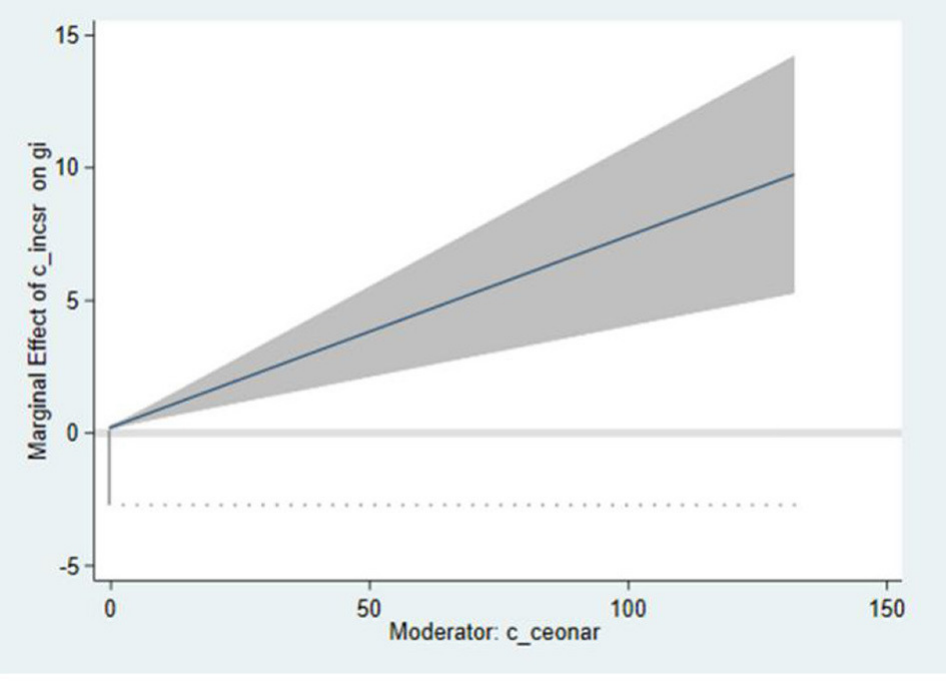
Interaction between c_incsr and c_ceonar.

In column (6) of Table 8, the effect of interact (cross-product terms of internal CSR and ceonar) on gti is positive and significant (β = 1.045, *p* < 0.001) and the main effect is also positive and significant. The results are consistent with the mixed effect regression, which provide strong support for Hypothesis 4. Figure 2 demonstrates the interaction between internal CSR and ceonar. The line represents the variation trend of internal CSR coefficient, and the shaded part represents confidence intervals. As shown in the figure, with the increase of CEO narcissism index, the internal CSR coefficient increases gradually, and the confidence intervals are above 0.

**Figure 3:**
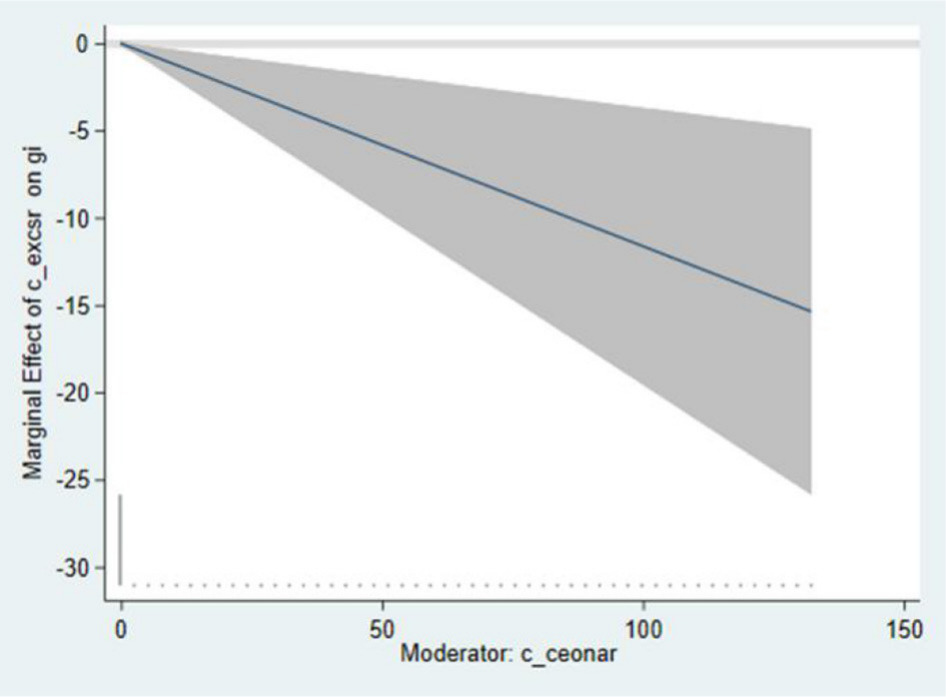
Interaction between c_excsr and c_ceonar.

In column (6) of Table 8, the effect of interact 1 (cross-product terms of external CSR and ceonar) on GTI is negative and significant (β = −1.515, *p* < 0.001) and the main effect is also negative and significant. The results are consistent with the mixed effect regression, which do not support Hypothesis 5. The reason may be that narcissistic CEOs take the external views on themselves seriously, and will take the initiative to use more of the resources of the company to maintain external relations and their own image. Thus, the resources for technology innovation are reduced, and the negative correlation between external CSR and green technology innovation is enhanced. Figure 3 demonstrates the interaction between external CSR and ceonar. The line represents the variation trend of external coefficient of CSR, and the shaded part represents confidence intervals. As shown in the figure, with the increase of CEO narcissism index, the external coefficient of the CSR increases negatively, and the confidence intervals are below 0.

### Robustness Check

To ensure the robustness of the results, this study tested the endogeneity of the models, changed the measurement method of CSR, and conducted time-lagged treatment of the effects of core independent variables (csr, incsr, and excsr) on GTI including the endogeneity test, main effect regression after replacing the measurement of csr (csr1), and core independent variables (csr, incsr, and excsr) with 1-year lag regression. The methods are detailed below:

#### Endogeneity Test

Corporate social responsibility, as an effective way for enterprises to obtain external resources and enhance corporate image, will promote GTI through the increase of R&D investment and innovation knowledge, while GTI, as an important initiative for firms to protect the environment, will enhance CSR. Thus there is a causal relationship between CSR and GTI. For this reason, in this paper, csr, incsr, and excsr with 1-year lag were selected as instrumental variables. The rationality of the instrumental variables is as follows: (1) L.csr, L.incsr, and L.excsr are significantly correlated with csr, incsr, and excsr; (2) Since the lagged variables have occurred, their values are fixed from the current period perspective. Therefore, instrumental variables are not correlated with the disturbance terms; (3) The GTI in the current period cannot affect the csr, incsr, and excsr in the past, so L.csr, L.incsr, and L.excsr are exogenous. In summary, L.csr, L.incsr, and L.excsr satisfy the correlation and exogeneity. Since this paper used panel data, the endogeneity test was conducted using XTIVREG, and the test results are shown in Table 9.

**Table 9:** Endogeneity test.

	**Underidentification**		**Weak identification**	**Overidentification**	**Endogeneity**	
L.csr	LM = 34.632	*p* = 0.000	F = 35.680	Sargan = 0	X2(1) = 0.002	*p =* 0.966
L.incsr	LM = 26.527	*p =* 0.000	F = 27.113	Sargan = 0	X2(1) = 0.038	*p =* 0.845
L.excsr	LM = 49.009	*p =* 0.000	F = 51.220	Sargan = 0	X2(1) = 0.031	*p =* 0.860

As shown in Table 9, all *P*-values of the underidentification test = 0.00, so the original hypothesis of the unidentifiable is strongly rejected; all F statistics of the Weak identification test are greater than 16.38, so its level of truthfulness cannot be greater than 10%, and the original hypothesis of weak instrumental variables is rejected; all Sargan-values of the overidentification test = 0, so there is no overidentification. In conclusion, three instrumental variables have reasonableness. *P*-values of endogeneity test are greater than 0.1, so the original hypothesis that all independent variables are exogenous cannot be rejected, so there is no serious endogeneity problem in this paper.

#### Change the Measurement Method of CSR

Drawing on the study of Li et al. (2018), this paper used the scores from Rankins CSR ratings (http://www.rksratings.cn) to measure the CSR and defined it as the core independent variable (csr1). In column (7) of Table 10, the effect of csr1 on gti is positive and significant (β = 0.358, *p* < 0.05) and still supports the Hypothesis 1.

**Table 10:** Results of robustness check.

	**(7)**	**(8)**	**(9)**
**Variables**	**gti**	**gti**	**gti**
csr1	0.385**		
	(2.54)		
Area	0.186**	−0.121***	−0.107***
	(2.35)	(−4.09)	(−3.62)
Nature	−0.077	−0.024	−0.007
	(−0.80)	(−0.39)	(−0.11)
Industry	0.191**	0.119*	0.120**
	(2.03)	(1.95)	(1.98)
Size	0.140***	0.106***	0.114***
	(4.98)	(5.52)	(6.03)
Profitability	−0.339***	−0.307***	−0.398***
	(−6.50)	(−3.34)	(−4.23)
Tsrat	0.124	0.050	0.060
	(0.35)	(0.31)	(0.37)
L.csr		0.004**	
		(2.20)	
L.incsr			0.018***
			(4.64)
L.excsr			−0.029***
			(−4.97)
Constant	−3.784***	−1.372***	−1.643***
	(−5.07)	(−3.18)	(−3.84)
Observations	785	1,329	1,329
R-squared	0.083	0.057	0.072
F test	0	0	0
r2_a	0.0743	0.0520	0.0668
F	15.28	11.41	12.89

***p < 0.01, **p < 0.05.

#### Time-Lagged Treatment of the Effects of Core Independent Variables (csr, incsr, and excsr) on gti

Considering the long-term nature of GTI, the effects of the core independent variables (csr, incsr, and excsr) on gti may have time lag. This study used the method of lagging core independent variables to solve. In column (8) of Table 10, the effect of csr with 1-year lag on gti is positive and significant (β = 0.004, *p* < 0.05). In column (9) of Table 10, the effect of incsr with 1-year lag on gti is positive and significant (β = 0.018, *p* < 0.001), and the effect of excsr with 1-year lag on gti is negative and significant (β = −0.029, *p* < 0.001). Thus, the Hypotheses 1, 2 and 3 are still supported.

From the test results, the fitting effect, variable sign, and the significance level of the two methods of changing the measurement method of CSR and the time-lagged treatment are consistent with the previous regression results, and the core independent variables (csr, incsr, and excsr) have no serious endogeneity, which preliminarily confirms the robustness of the statistical results.

## Conclusions and Implications

### Conclusion

Since the outbreak of the COVID-19 pandemic in 2019, human lives, trade, economy, and businesses across the globe have been threatened. Many scholars have emphasized the importance of studying the green behaviors of the firms in the context of dramatic, social, and economic change. Therefore, this study aims to identify the impact of corporate social responsibility (CSR) on the green technology innovation (GTI) practices with the moderating effect of the chief executive officer (CEO) narcissism on the basis of the upper echelons theory and stakeholder theory.

**Figure 4:**
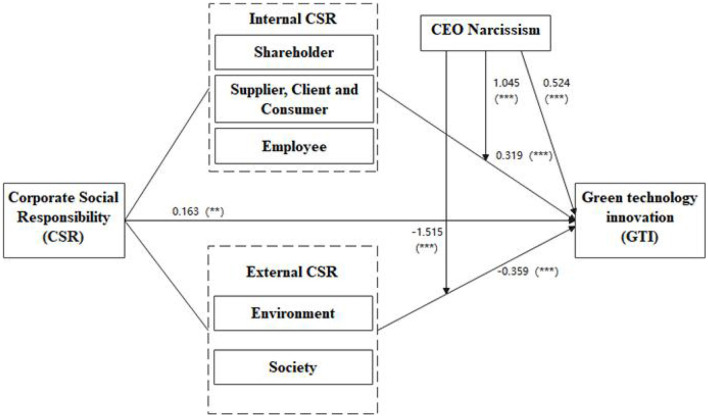
Structural model of the study.

As exhibited in Figure 4, six hypotheses were constructed in the study. Among them, four were direct hypotheses, and two were proposed for the moderation effect. The empirical results show that: (1) The fulfillment of CSR by a firm has a significant positive impact on GTI. (2) The fulfillment of internal CSR has a significant positive impact on GTI. (3) The external CSR has a significant negative impact on GTI. The more a firm fulfills its external CSR, the more dispersed its resources will be, and its focus will be shifted from improving the product quality and promoting technological innovation to social relationship maintenance. In China, many valuable public resources including land, environment, state-owned assets, financial subsidies, and government credit are in the hands of the governments at different levels, which are more powerful in allocating resources. As such, the more political resources a firm has, the more likely the performance of the firm is improved through rent-seeking rather than innovation activities. External CSR is mainly responsible to meet the demands of the government, community, and other organizations that are not directly involved in the production and operation. Politically motivated CEOs engage in external CSR activities in order to establish good connections with the government and other key external stakeholders (Yang, 2018). To obtain preferential policies, such as market access, tax incentives, and government subsidies, narcissists are likely to engage in excessive external CSR activities to obtain government trust. As such, a considerable amount of resources are spent on these external CSR activities, resulting in the reduction of input in GTI (Zhang et al., 2016). (4) CEO narcissism has a significant positive impact on GTI; (5) CEO narcissism positively moderates the impact of internal CSR on GTI. (6) CEO narcissism puts great emphasis on external relations and the corporate image rather than technological innovation and it negatively moderates the impact of external CSR on GTI. A firm prioritizes the demands of its stakeholders based on the salience of stakeholders, which in turn depends on the power of the stakeholder, legitimacy, and urgency (De and Swaen, 2016). Highly narcissistic CEOs are particularly eager to seek the attention and recognition of key external stakeholders, such as government and public media, which can bring them greater social popularity. Therefore, they are likely to pay more attention to externally-oriented CSR activities than the internal claims of the stakeholders. CEOs who are narcissistic will commit greater time and resources to external CSR owing to the fact that it would generate media praise and government attention (Tang et al., 2015). Given that narcissistic CEOs are more concerned with the opinions of the key external stakeholders and the maintenance of the relationship with them, they have a tendency to place greater emphasis on external CSR even when the financial performance of the firms is poor compared to their less narcissistic peers (Chin et al., 2013). Such external CSR serves as a means employed by CEOs to reinforce self-image, strengthen the relationship with key external stakeholders, and fulfill their personal needs. The CEOs tend to devote more resources to external CSR with accompanying reduction of the vital resources for innovation. Therefore, the narcissism of the CEOs strengthens the negative relationship between external CSR and GTI.

### Practical Implications

Based on the theory developed and empirical evidence obtained, the study suggests the following practical implications.

First, given the potential benefits of CSR, firms may actively engage in CSR activities. This study finds that internal CSR plays an important role in enhancing the motivation of the employees and satisfying the interests of shareholders, suppliers, and customers, thus creating a favorable internal setting for GTI. GTI contributes balance environmental protection and economic development, which is a key to create a sustainable society (Rennings et al., 2016; Sun et al., 2008). Therefore, there is the need for the active engagement of the firms in internal CSR and build up and maintain good relationships with market stakeholders, such as employees, shareholders, suppliers, and customers. However, due to the motive of the firms for garnering more attention from the government and public, external CSR is merely a proven means to gain more market share instead of the sustainable development of the society. It suggests the need to develop more rigorous governance mechanisms to ensure that the external CSR is to benefit and contribute to the welfare of the society as a whole, not just to increase the profitability of businesses.

Second, although narcissism is a dark side of the personality trait (Al-Shammari et al., 2019), it leads to both positive and negative effects. On the positive side, the focus of the narcissistic CEOs on internal CSR can strengthen the ties of the CEOs with employees, suppliers, customers, and shareholders, which in turn will promote the GTI. However, if narcissistic CEOs focus on external CSR for the purpose of seeking attention and praise, it is likely that they will shift their attention more to fostering the relationship with the government and other civic institutions instead of the innovation-oriented development of the firms. Therefore, the study results suggest the firms need to look more closely at the narcissistic trait of CEOs. On the one hand, the narcissistic CEOs should be encouraged to make their commitment toward employees, shareholders, suppliers, and customers. On the other hand, the behaviors aiming at generating greater attention and personal fame when engaging in CSR should be limited.

### Limitations and Further Research

This paper investigates the impact mechanism of CSR on firm GTI from the theoretically developed and empirical evidence. There are still some limitations for further exploration. First, this paper only conducted one lag period to address the lag of GTI. Given the different performance and life cycles of R & D, the number of lag periods is not the same in different firms. Thus this study can only conduct a lag operation in general terms without considering the specific features of each firm, which will influence the results of the research and fail to provide effective advice for firms to conduct GTI. This issue should be improved in future studies. Second, this study measures CEO narcissism indirectly due to the difficulties in China in using National Provider Identifier (NPI) for research questionnaires to measure CEO narcissism directly. Owing to the fact that CEOs in China are more reluctant to disclose information than their western peers, it is hard to obtain enough questionnaire responses and accompanying sufficient data to support the study. Therefore, we employ five measures developed from the four dimensions of the NPI to measure CEO narcissism indirectly. Although this method can remedy the defect of data unavailability, it could cause a certain degree of bias in our research. For example, it may not reflect the true intentions of the CEOs. We encourage future scholars to conduct a study measuring CEO narcissism directly using the NPI when the necessary data become more available.

## Data Availability Statement

The data analyzed in this study is subject to the following licenses/restrictions: As the datasets is non-public, readers can request it from the corresponding author. Requests to access these datasets should be directed to Xiangjiao Shi, s1641152616@163.com.

## Author Contributions

HY was responsible for writing the theoretical part of the article, as supervisor in the research process, and provides financial support for this study as the research project leader. XS was responsible for the data collection and writing of the empirical research part of the article. SW was responsible for guiding the research methodology and supervising the writing of the empirical part. All authors contributed to the article and approved the submitted version.

## Funding

This research was underwritten by grants from the Social Science Funding Program, Shandong Province, China. Program number: 21CGLJ27.

## Conflict of Interest

The authors declare that the research was conducted in the absence of any commercial or financial relationships that could be construed as a potential conflict of interest.

## Publisher's Note

All claims expressed in this article are solely those of the authors and do not necessarily represent those of their affiliated organizations, or those of the publisher, the editors and the reviewers. Any product that may be evaluated in this article, or claim that may be made by its manufacturer, is not guaranteed or endorsed by the publisher.
